# Factors affecting the Apgar score of offsprings born to mothers suffering from systemic lupus erythematosus

**DOI:** 10.1097/MD.0000000000022843

**Published:** 2020-10-23

**Authors:** Hiromi Shimada, Tomohiro Kameda, Kenji Kanenishi, Nobuyuki Miyatake, Shusaku Nakashima, Risa Wakiya, Mikiya Kato, Taichi Miyagi, Mai Mahmoud Fahmry Mansour, Toshiyuki Hata, Norimitsu Kadowaki, Hiroaki Dobashi

**Affiliations:** a Department of Internal Medicine, Division of Hematology, Rheumatology and Respiratory Medicine; b Department of Perinatology and Gynecology; c Department of Hygiene, Faculty of Medicine, Kagawa University, Kagawa, Japan.

**Keywords:** anti-double-stranded DNA antibody, Apgar score, pregnancy, systemic lupus erythematosus

## Abstract

To reveal which disease activity parameters affect low Apgar scores of newborns, which is considered as a predictive parameter for neurological development.

We examined retrospectively the data from 42 newborns who were delivered from systemic lupus erythematosus (SLE) mothers from 2006 to 2019. We evaluated whether the disease activity parameters, such as the achievement ratio of lupus low disease activity state (LLDAS), SLE disease activities index (SLEDAI), complement level, titer of anti-double stranded DNA (anti-dsDNA) antibody, therapeutic agents were related with low Apgar scores of newborns.

In 42 newborns, adverse pregnancy outcomes, especially preterm birth (16.7%), low birth weight (31.0%) light-for-date (11.9%) were associated with disease activity parameters or prednisolone dose. Apgar scores at 1 minute were related with unachieved LLDAS and the titer of anti-dsDNA antibody at first and third trimester, SLEDAI score and complement level at third trimester, mean prednisolone dose. Apgar scores at 5 minutes were also associated with the titer of anti-dsDNA antibodies at first and third trimester and mean prednisolone dose. Multivariate analysis showed only high titer of anti-dsDNA antibody was significantly associated with low Apgar score at both one minute and 5 minutes.

In our retrospective study, high titer of anti-dsDNA antibodies at first and third trimester was a risk factor for low Apgar scores of newborns born to SLE mothers. We considered that high titer of anti-dsDNA antibody influenced on childrens neurological development, therefore, there is a need for long-term follow-up study of SLE offsprings.

## 1 Introduction

Systemic lupus erythematosus (SLE) is an autoimmune disease that occurs in women of childbearing age. The disease can vary from mild to life-threatening, with a variety of symptoms including rash, arthritis, anemia, thrombocytopenia, serositis, nephritis, seizures, and psychosis. As the treatment strategy for SLE has improved considerably, more SLE women are able to become mothers. However, SLE women have high risks for adverse pregnancy outcomes (APOs) including preterm birth, intrauterine growth retardation, and pregnancy loss.^[1–3]^ Previous studies have found that high disease activity, hypocomplementemia, the presence of anti-double stranded DNA (anti-dsDNA) antibodies and antiphospholipid antibodies, lupus nephritis, and corticosteroid dose were risk factors for APOs.^[4–8]^

On the other hand, many SLE women hope healthy growth and development of their children after birth, and short and long-term outcome of children born to SLE mothers is most important issue. However, it remains unclear whether maternal SLE influences their childrens long-term general health and development. Some reports have revealed associations between maternal SLE and/or antiphospholipid syndrome and neurological abnormalities including learning disorders (especially dyslexia) and autism spectrum disorders (ASD) among the children born to SLE mothers.^[9–12]^ Vient et al reported that children born to SLE women were more frequently found to have a diagnosis of ASD compared with control (frequency of ASD was 1.4% vs 0.6%).^[10]^ However, it is unknown which factors influence neurological disorders on these children.

Apgar score is used as a common clinical index for assessing neonatal health status soon after birth. It evaluates heart rate, respiration, color, muscle tone, and reflex, and determines whether extra medical care or emergency care is needed. The test is performed at 1 minute and 5 minutes after birth. Apgar score at 5 minutes is demonstrated as newborns neurological development. Several studies have demonstrated associations between low Apgar score and neurological, cognitive, and psychological abnormalities.^[13–15]^ In particular, low Apgar score at 5 minutes has been shown to be associated with increased risks of postnatal mortality, cerebral palsy, seizure, mental retardation, and autism spectrum disorders.^[13–17]^ However, it has never been reported the association between maternal SLE including its disease activity index and low Apgar score. Therefore, we consider it is important to investigate the relationship between Apgar score and maternal SLE and to identify predictive factors for low Apgar score among births from women with SLE.

The aim of our study was to identify the predictive parameters for low Apgar score among newborns who were delivered from SLE mothers in our institution.

## 2 Material and methods

### 2.1 Patients and data collection

Our study was retrospective observational study. SLE patients and their newborns were enrolled, who were delivered at Kagawa University Hospital from May 2006 to September 2019. We excluded the case of miscarriage, and all cases of live birth were intended for our study. These patients were diagnosed with SLE according to the 1997 revision of the 1982 American College of Rheumatology criteria for the classification of SLE. We extracted the data on 42 SLE patients who were treated in our institution from preconception counseling to pregnancy and delivery. We investigated these womens clinical background characteristics (age at conception and disease duration defined as the period from disease onset to conception), autoantibody profiles (anti-SS-A/B antibodies, anticardiolipin antibodies, anti-β2 glycoprotein I antibody and lupus anticoagulant), SLE disease activity scores and their components, therapeutic agents before and during pregnancy. The association between these parameters and pregnancy outcomes, which included gestational weeks at delivery, preterm birth, birth weight, light-for-date (LFD) and Apgar score of newborns, was evaluated. Apgar score was assessed through measures of appearance (color), pulse (heartrate), grimace (reflex), activity (muscle tone), and respiration.^[18]^ Gynecologist or pediatrician scored each index from 0 to 2 and calculated all of these scores. LFD was defined as birth weight of the newborn lower than the 10th percentile.

All data were collected from medical records at Kagawa University Hospital. Disease activity score was calculated using the SLE Disease Activity Index (SLEDAI),^[19,20]^ which is scored from 0 to 105 and includes 24 weighted objective clinical and laboratory variables, for example, neurological dysfunction, arthritis, rash, proteinuria, low complement, and high titer of anti-dsDNA antibodies. In addition, disease activity was also evaluated by lupus low disease activity state (LLDAS), which was defined as^[1]^ SLEDAI-2≦4 with no activity in major organ systems (renal, central nervous system, cardiopulmonary vasculitis, fever) no hemolytic anemia or gastrointestinal activity,^[2]^ no new lupus disease activity compared with the previous assessment,^[3]^ physician global assessment (scale 0–3)≦1,^[4]^ a current prednisolone dose≦7.5 mg per day, and^[5]^ well tolerated standard maintenance doses of immunosuppressive drugs and approved biologic agents.^[21]^ The components of SLE disease activity score included complement level; C3, C4, and CH50 and the titer of anti-dsDNA antibody. All laboratory tests were performed using standard methods.

Our research was retrospective observational study, therefore, we have never received the ethical approval. We did not obtain patients informed consents, however, we disclosed the information of our research.

### 2.2 Statistical analysis

Values are presented as mean ± standard deviation for continuous variables and as numbers (percentage) for categorical variables. We investigated the association with univariate and multivariate logistic regression analysis between the Apgar score of newborns and the SLEDAI score, LLDAS achievement, disease activity components, and glucocorticoid dose during pregnancy. Descriptive statistics were compared using the Wilcoxon signed-rank test for continuous variables, and Fishers exact test for categorical variables. The correlation analysis was performed by Spearman Correlation Test. *P* value of <.05 was considered significant. We considered parameters with significant differences to be predictive factors. All analyses were conducted using JMP for Mac, Version 13.0.0 (SAS institute, Japan).

## 3 Results

### 3.1 Patients characteristics at conception

There were 52 SLE pregnant patients in study period, and 10 cases were miscarriage, which were excluded. Therefore, our study used the data from 42 SLE pregnant patients and their newborns. The patients characteristics at conception are shown in Table 1. The mean age at delivery was 32.2 ± 4.2 years old, and mean disease duration was 10.8 ± 5.3 years. More than half of all cases were positive for anti-SS-A antibody (64.3%) and antiphospholipid antibody (50.0%).

**Table 1:**
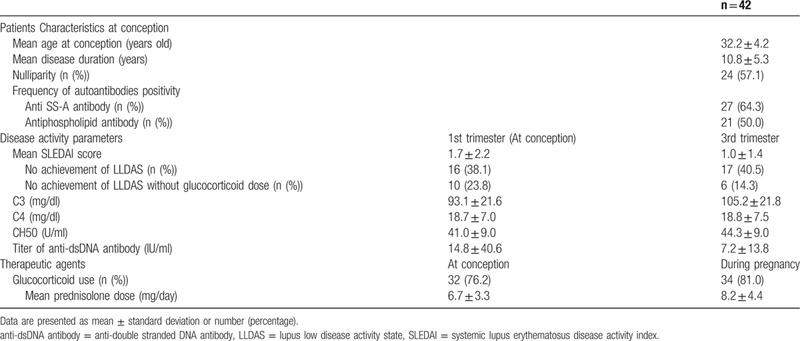
Patients characteristics at conception and disease activity parameters, therapeutic agents.

### 3.2 Disease activity parameters and therapeutic agents

Table 1 also showed disease activity parameters and therapeutic agents. The mean SLEDAI score in the first and third trimesters was 1.7 ± 2.2 and 1.0 ± 1.4, respectively. Fourteen cases (33.3%) had disease flare which was defined as more than 1 increase of SLEDAI score, and mean gestational weeks of disease flare was 14.2 ± 9.8 weeks. Sixteen cases (38.1%) did not achieve LLDAS and 10 cases (23.8%) did not achieve that without glucocorticoid dose at conception. Complement level was normal (C3; 93.1 ± 21.6, C4; 18.7 ± 7.0, CH50; 41.0 ± 9.0), however, the titer of anti-dsDNA antibody was a little high (14.8 ± 40.6). Glucocorticoid was administered in about 80% cases at the conception and during pregnancy. Mean prednisolone dose was 6.7 ± 4.4 mg per day at the conception and 8.2 ± 4.4 mg per day during pregnancy. The mean dose during pregnancy was increased compared with that of conception.

### 3.3 Adverse pregnancy outcomes is associated with disease activity parameter

Pregnancy outcomes are shown in Table 2. In 42 cases, Cesarean section was performed in 10 cases (23.8%). The mean gestational time was 37.9 ± 2.7 weeks, and preterm birth occurred in seven cases (16.7%). High titer of anti-dsDNA antibody every time (at first or third trimester), and unachieved LLDAS at third trimester were related with preterm birth significantly (*P* = .04, 0.01, and <0.05, respectively, Supplemental table 1). Hypertensive disorders of pregnancy occurred in 3 cases. There were also no significant differences on any disease activity parameters between the cases of hypertensive disorder and the others (data not shown).

**Table 2:**
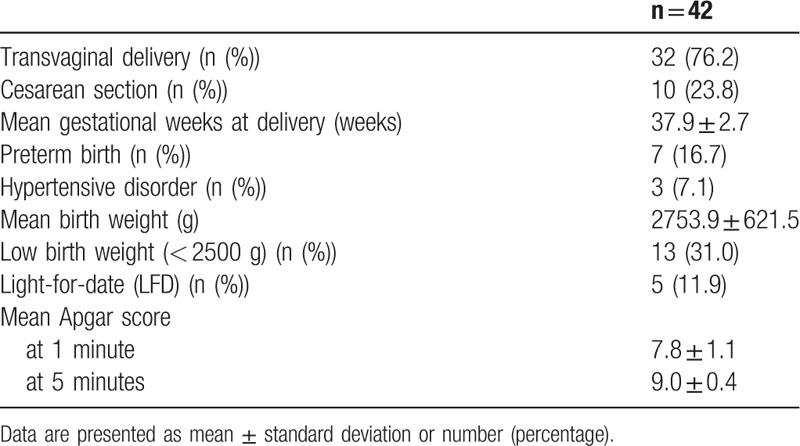
Pregnancy outcome.

As for the outcomes of newborns, the mean birth weight was 2753.9 ± 621.5 g. Thirteen newborns were low birth weight (less than 2500 g). In their mothers, hypocomplement of C3 every time (at first or third trimester) and CH50 at first trimester, in addition, higher rate of glucocorticoid use were observed (Supplemental Table 2). Five newborns (11.9%) were LFD, and hypocomplement of CH50 at first trimester, and high SLEDAI score at third trimester was detected in LFD mothers (Supplemental Table 3).

### 3.4 Apgar score is associated with the titer of anti-dsDNA antibody

As for Apgar score of newborns, mean Apgar score at 1 minute and 5 minutes was 7.8 ± 1.1 and 9.0 ± 0.4, respectively. Tables 3 and 4 shows the results of the univariate analysis for Apgar score at 1 minute and 5 minutes. Apgar score at 1 minute was correlated with unachieved LLDAS and the titer of anti-dsDNA antibody at first and third trimester. SLEDAI score, complement level at third trimester, and mean prednisolone dose during pregnancy was also associated with that. Apgar score at 5 minutes was correlated with only the titer of anti-dsDNA antibodies at first and third trimester and mean prednisolone dose during pregnancy. Tables 5 and 6 shows multivariate analysis of relevance to low Apgar score at 1 minute and 5 minutes. We demonstrated that only high titer of anti-dsDNA antibody was significantly associated with low Apgar score at both 1 minute and 5 minutes. In our study, explanatory variable was limited by small number of cases, so that we evaluated with several combinations of variables. Other disease activity index and treatment agent was not associated with Apgar score. In addition, birth weight of newborns was also correlated with Apgar score at both 1 minute and 5 minutes in univariate analysis. However, the titer of anti-dsDNA antibody was more significant association with Apgar score in multivariate analysis (data not shown).

**Table 3:**
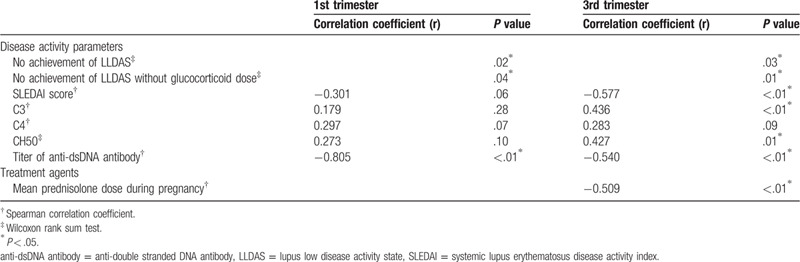
Univariate analysis of relevance to low Apgar score at 1 minute.

**Table 4:**
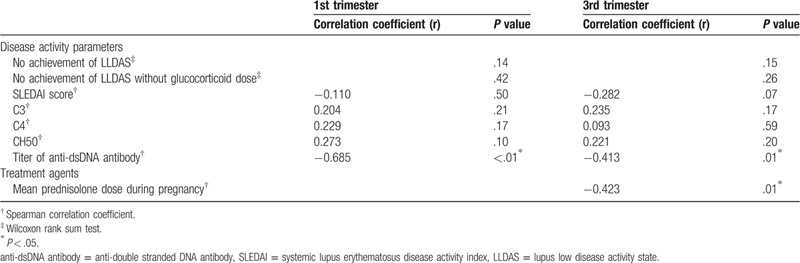
Univariate analysis of relevance to low Apgar score at 5 minute.

**Table 5:**
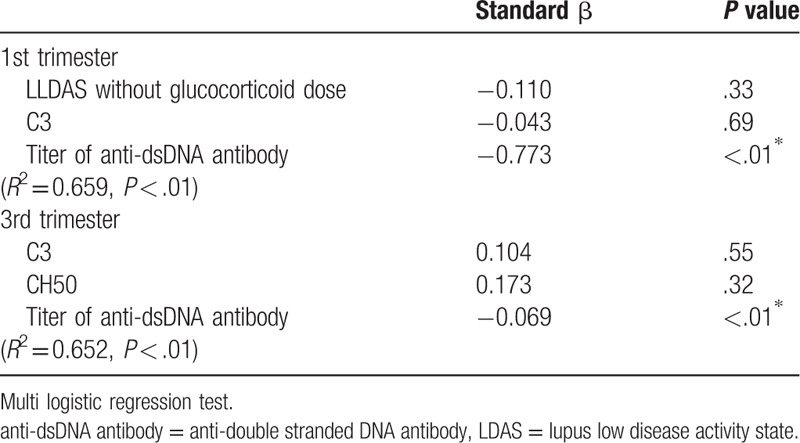
Multivariate analysis of relevance to low Apgar score at 1 minute.

**Table 6:**
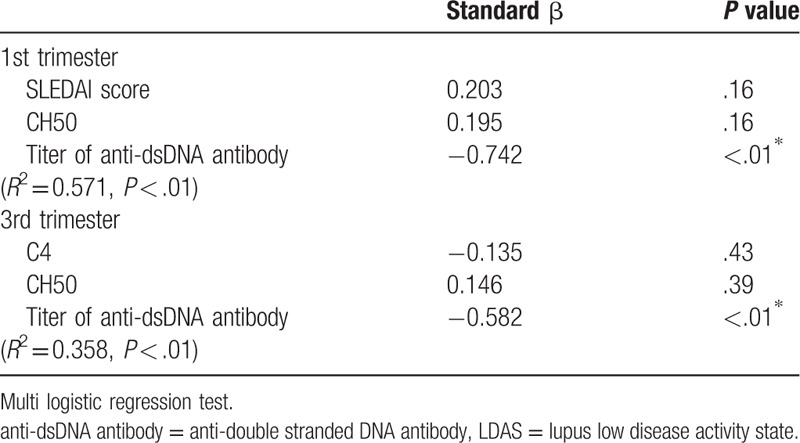
Multivariate analysis of relevance to low Apgar score at 5 minute.

## 4 Discussion

Our retrospective study identified the association between low Apgar score of newborns and high titer of anti-dsDNA antibodies at both first and third trimester. In the univariate analysis, no achievement of LLDAS and the anti-dsDNA antibody at first and third trimester, SLEDAI score and complement level at third trimester, and mean prednisone dose during pregnancy were significantly related to Apgar score at 1 minute, and the titer of anti dsDNA antibody at first and third trimester and mean prednisolone dose during pregnancy were also significantly related with Apgar score at 5 minutes. Furthermore, in multivariate analysis, only high titer of anti-dsDNA antibody was significantly associated with low Apgar score at both 1 minute and 5 minutes. Other disease activity parameters were not associated with low Apgar score.

Apgar scores is widely used as a common clinical index for assessing neonatal health status after birth. Especially, Apgar score at 5 minutes is demonstrated as a predictive index for neurological, cognitive, and psychological abnormalities of newborns.^[13–15]^ In addition, some studies have shown that low Apgar score at 5 minutes has been shown to be associated with increased risks of postnatal mortality, cerebral palsy, seizure, mental retardation, and autism spectrum disorders.^[13–17]^ Recently, several reports revealed that maternal SLE and/or antiphospholipid syndrome has been found to influence neurological development—especially learning disorders and autism spectrum disorders—among these womens children.^[9–12]^ In these reports, antiphospholipid and anti SS-A antibodies have been identified as risk factors for these disorders. In our study, these autoantibodies were not associated with low Apgar score (data not shown). However, our data revealed that only high titer of anti-dsDNA antibody was significantly related with low Apgar score in univariate and multivariate analysis. Anti-dsDNA antibody is full-body antibody with Fc receptor, which could transfer placenta. It is necessary to consider some negative influence to fetus by placenta-transferred anti-dsDNA antibody. Actually, the titer of anti-dsDNA antibody of fetus could not be measured in our study, however, we speculate that anti-dsDNA antibody might transfer to fetus across the placenta and give negative influence to fetus.

Unfortunately, we were unable to follow the neurological development of children born to SLE mothers in our sample. However, because Apgar sore is strongly associated with neurological development, we consider that high titer of anti-dsDNA antibody gives major impact on neurological development of SLE offsprings through low Apgar score. This might be related with the high frequency of neurological disorders such as learning disorders and autism spectrum disorders of SLE offsprings.

Our study had several limitations. First, our sample comprised a small number of patients, and there were also a small number of outcome events because of the single center study, which might have resulted in low statistical power. Second, because this was a retrospective study, we could not fully exclude selection or information bias, and some cases lacked data such as laboratory findings. Third, this study was conducted in only one expert institution; therefore, we might have collected data on patients with relatively high levels of disease activity and high risks for pregnancy.

## 5 Conclusion

In our retrospective study, low Apgar scores of newborns who were delivered from SLE mothers were associated with high titer of anti-dsDNA antibodies at first and third trimester in both univariate and multivariate analysis. Thus, high titer of anti-dsDNA antibodies might be a predictive factor for low Apgar scores which are associated with neurological development of newborns. In preconception counseling, it is important for our rheumatologists to explain these predictive factors clearly to SLE patients who hope to conceive. It will be beneficial for both SLE mothers and their newborns to manage those factors carefully before and during pregnancy. Furthermore, there is a need for long-term follow-up studies focusing on the neurological development of children born to SLE mothers.

## Acknowledgments

We thank Jennifer Barrett, PhD, from Edanz Group (www.edanzediting.com/ac) for editing a draft of this manuscript.

## Author contributions

**Conceptualization:** Hiroaki Dobashi.

**Data curation:** Hiromi Shimada, Nobuyuki Miyatake.

**Formal analysis:** Hiromi Shimada, Nobuyuki Miyatake.

**Investigation:** Hiromi Shimada.

**Methodology:** Hiromi Shimada, Kenji Kanenishi, Nobuyuki Miyatake, Hiroaki Dobashi.

**Supervision:** Tomohiro Kameda, Toshiyuki Hata, Norimitsu Kadowaki, Hiroaki Dobashi.

**Validation:** Kenji Kanenishi, Nobuyuki Miyatake, Norimitsu Kadowaki, Hiroaki Dobashi.

**Visualization:** Nobuyuki Miyatake.

**Writing – original draft:** Hiromi Shimada.

**Writing – review & editing:** Tomohiro Kameda, Kenji Kanenishi, Shusaku Nakashima, Risa Wakiya, Mikiya Kato, Taichi Miyagi, Mai Mahmoud Fahmry Mansour, Hiroaki Dobashi.

## Supplementary Material

Supplemental Digital Content

## Supplementary Material

Supplemental Digital Content

## Supplementary Material

Supplemental Digital Content
