# Supplementary material for: Factors affecting the Apgar score of offsprings born to mothers suffering from systemic lupus erythematosus
Source: Medicine (Baltimore). 2020 Oct 23;99(43):e22843. doi: 10.1097/MD.0000000000022843 (PMC7581183; doi:10.1097/MD.0000000000022843)
Supplement: Supplemental Digital Content [file medi-99-e22843-s001.pptx]

## Slide 1
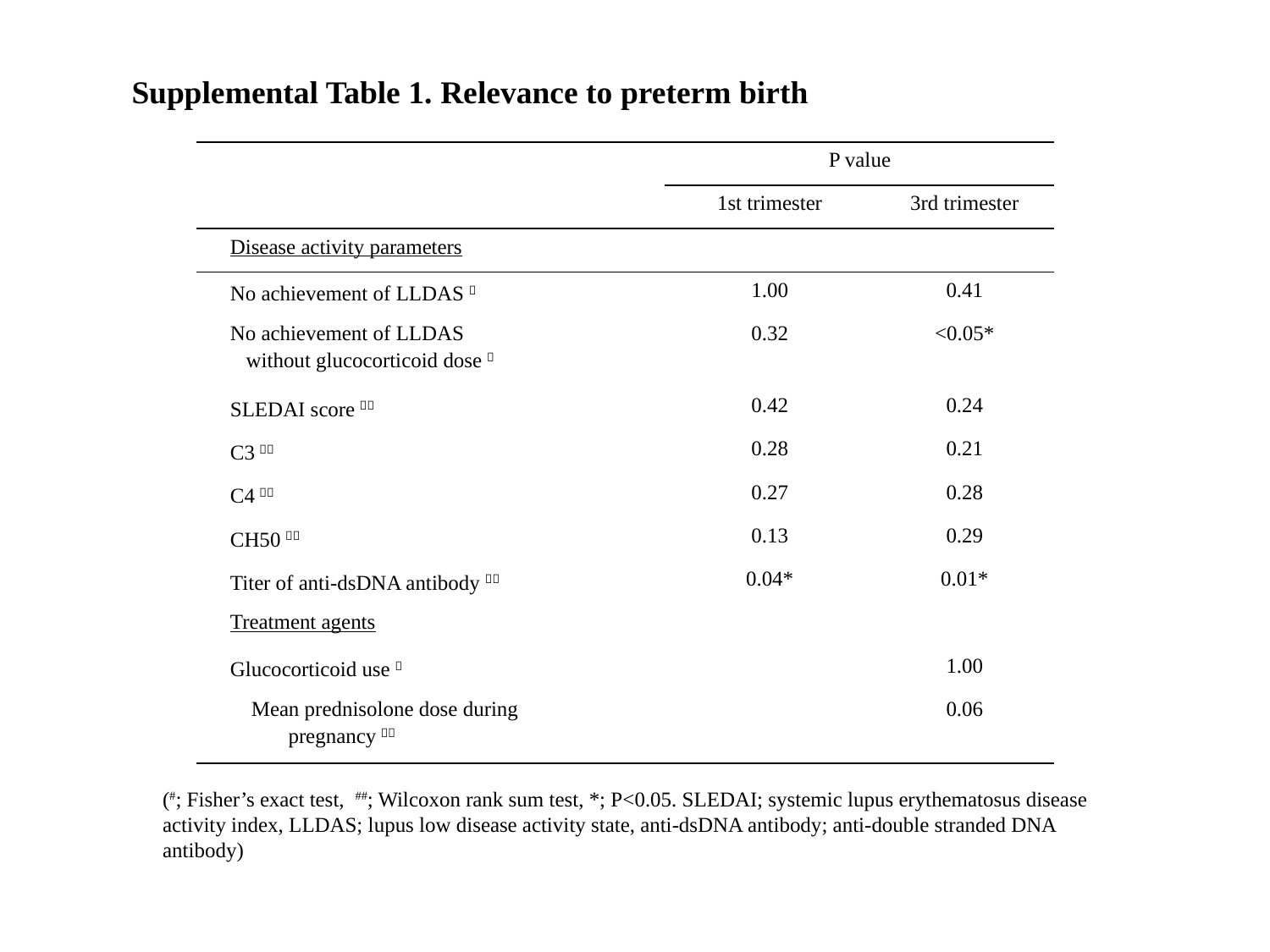

Supplemental Table 1. Relevance to preterm birth
| | P value | |
| --- | --- | --- |
| | 1st trimester | 3rd trimester |
| Disease activity parameters | | |
| No achievement of LLDAS＃ | 1.00 | 0.41 |
| No achievement of LLDAS without glucocorticoid dose＃ | 0.32 | <0.05\* |
| SLEDAI score＃＃ | 0.42 | 0.24 |
| C3＃＃ | 0.28 | 0.21 |
| C4＃＃ | 0.27 | 0.28 |
| CH50＃＃ | 0.13 | 0.29 |
| Titer of anti-dsDNA antibody＃＃ | 0.04\* | 0.01\* |
| Treatment agents | | |
| Glucocorticoid use＃ | | 1.00 |
| Mean prednisolone dose during pregnancy＃＃ | | 0.06 |
(#; Fisher’s exact test, ##; Wilcoxon rank sum test, *; P<0.05. SLEDAI; systemic lupus erythematosus disease activity index, LLDAS; lupus low disease activity state, anti-dsDNA antibody; anti-double stranded DNA antibody)
